# Unlocking the potential for microbiome‐based therapeutics to address the sustainable development goal of good health and wellbeing

**DOI:** 10.1111/1751-7915.70041

**Published:** 2024-11-02

**Authors:** Emily L. Gulliver, Sara K. Di Simone, Michelle Chonwerawong, Samuel C. Forster

**Affiliations:** ^1^ Centre for Innate Immunity and Infectious Diseases Hudson Institute of Medical Research Clayton Victoria Australia; ^2^ Department of Molecular and Translational Science Monash University Clayton Victoria Australia; ^3^ Ritchie Centre, Hudson Institute of Medical Research Melbourne Victoria Australia; ^4^ Department of Paediatrics Monash University Melbourne Victoria Australia

## Abstract

Recent years have witnessed major advances and an ever‐growing list of healthcare applications for microbiome‐based therapeutics. However, these advances have disproportionately targeted diseases common in high‐income countries (HICs). Within low‐ to middle‐income countries (LMIC), opportunities for microbiome‐based therapeutics include sexual health epidemics, maternal health, early life mortality, malnutrition, vaccine response and infectious diseases. In this review we detail the advances that have been achieved in microbiome‐based therapeutics for these areas of healthcare and identify where further work is required. Current efforts to characterise microbiomes from LMICs will aid in targeting and optimisation of therapeutics and preventative strategies specifically suited to the unmet needs within these populations. Once achieved, opportunities from disease treatment and improved treatment efficacy through to disease prevention and vector control can be effectively addressed using probiotics and live biotherapeutics. Together these strategies have the potential to increase individual health, overcome logistical challenges and reduce overall medical, individual, societal and economic costs.

## Graphical abstract

Within low‐ to middle‐income countries (LMIC) opportunities for microbiome‐based therapeutics include sexual health epidemics, maternal health, early life mortality, malnutrition, vaccine response and infectious diseases. In this review we detail the advances that have been achieved in microbiome‐based medicines for these areas of healthcare and identify places where further work is required.

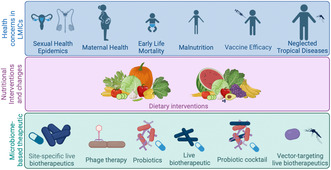

## INTRODUCTION

**FIGURE 1:**
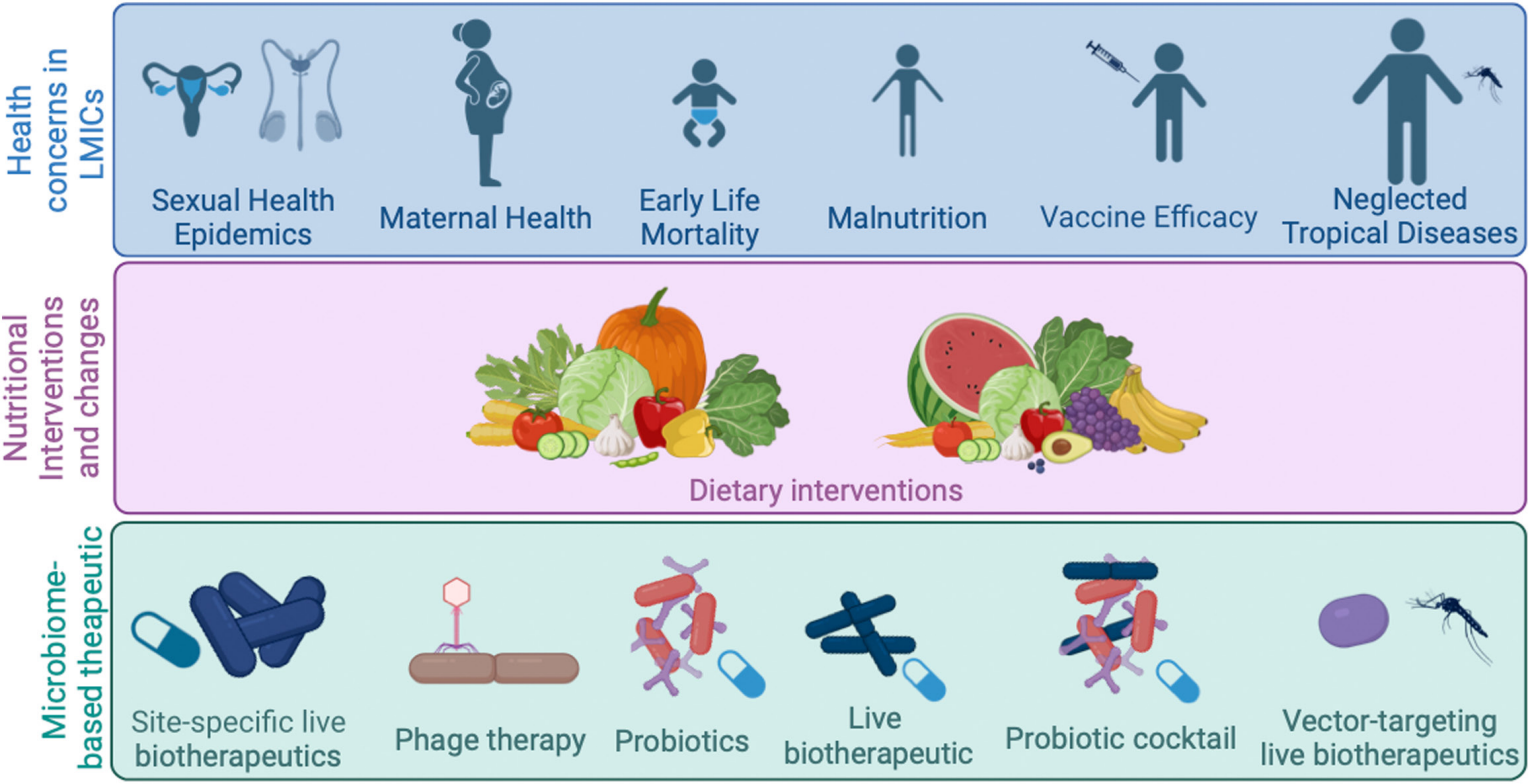
Areas where microbiome‐based therapeutics would benefit LMICs (blue box), with the corresponding nutritional interventions (purple box) and microbiome‐based therapeutic (green box) for each area.

The United Nations has outlined 17 goals for Sustainable Development, where the third goal is good health and well‐being, specifically aiming to ensure healthy lives and promote well‐being for all at all ages (UnitedNations, 2024). Given the role of the microbiome in health (Baunwall et al., 2020; Gill et al., 2018; Henn et al., 2021), microbiome‐based therapeutics may be a key tool to achieve this goal. A plethora of factors including diet, exercise, sex, age and illness can directly impact microbiome composition (Ghosh et al., 2022; Monda et al., 2017; Patangia et al., 2022). Numerous studies have now demonstrated the potential of microbiome biomarkers to diagnose and track disease (Metwaly et al., 2022; Qin et al., 2014; Ren et al., 2019; Zackular et al., 2014; Zwezerijnen‐Jiwa et al., 2023) and of microbiome‐based therapeutics as novel treatments (Baunwall et al., 2020; Gill et al., 2018; Henn et al., 2021). While microbiome‐based therapeutics have advanced rapidly in recent years, these developments have predominantly focused on diseases more prominent in high‐income countries (HICs) including inflammatory bowel disease (Bethlehem et al., 2024), graft‐versus‐host disease (Mathewson et al., 2016) and cancer immunotherapies (Matson et al., 2021). This review explores the current applications and future opportunities for microbiome‐based therapeutics in treating sexual health epidemics, maternal health, early life mortality, malnutrition, vaccine efficacy and infectious diseases, which disproportionately impact low‐ to middle‐income countries (LMIC; Figure 1).

## VARIATION IN MICROBIOMES BETWEEN LMICs AND HICs

Factors from diet and exercise (Monda et al., 2017; Sonnenburg et al., 2016) to access to sanitation (Cumming et al., 2019), clean drinking water (Fuhrmeister et al., 2023) and healthcare (Tozzo et al., 2022) can have dramatic impacts on microbiome composition and frequently vary across LMIC and HICs. Comparisons of the microbiomes of the hunter‐gatherer Hazda tribe with those in Western nations (Fragiadakis et al., 2019) have demonstrated clear seasonal variation and presence of bacterial families such as Prevotellaceae, Succinovibrionaceae, Paraprevotellaceae and Spirochaetaceae that are predominantly absent in the western microbiomes (Fragiadakis et al., 2019). Furthermore, work in Peru identified *Treponema* as a characteristic feature of hunter‐gatherer microbiomes (Obregon‐Tito et al., 2015). However, the hunter‐gatherer lifestyle is a single example of potential variations. Urban environments and sanitation, diet and malnourishment and incidence of infectious disease also vary across LMIC and HICs, and further characterisation of the microbiome in this context is required (Browne et al., 2024) to enable development of novel microbiome‐based therapeutics, targeted specifically for these groups.

## SEXUAL HEALTH EPIDEMICS

Over 90% of all new curable sexually transmitted infections (STIs) occur in LMICs (WHO, 2021) and 52% of all people living with human immunodeficiency virus (HIV) are located in eastern and southern Africa (Nyemba et al., 2021; UNAIDS, 2024), with a reported incidence of 20.8 million people, compared to 2.3 million in western and central Europe and North America (HIV.gov, 2024). Even for conditions with effective vaccines such as human papillomavirus (HPV) (Wigle et al., 2013) which also occur at higher frequency in LMIC (Nyemba et al., 2021; UNAIDS, 2024), it is still important to broaden the treatment strategy as these vaccines are expensive to manufacture and require cold chain for transportation (Akumbom et al., 2022; Wigle et al., 2013). The ability to culture and taxonomically identify the microbiome in specific tissue sites has improved our understanding of the vaginal microbiome on modulating human health and disease associated with the female genital tract (Baud et al., 2023; Juliana et al., 2021). Vaginal microbiome communities are typically dominated by lactic acid–producing, low pH‐tolerant *Lactobacillus* species, which are assigned to one of four major community sequence types (CSTs) (Baud et al., 2023; Juliana et al., 2021). Given the wealth of data supporting the role of the vaginal microbiome in many STIs, including HIV (Bayigga et al., 2019; Lebeau et al., 2022; Liu et al., 2022; Price et al., 2022), potential applications and targets for microbiome‐based therapeutics are numerous. Already, it is known that the presence of *Lactobacillus* species in the vaginal microbiome can reduce STI incidence including the presence of *Lactobacillus crispatus* decreasing the incidence of Chlamydia (Nardini et al., 2016) and a strain of *Lactobacillus jensenii* is being developed as a live biotherapeutic for HIV. This *L. jensenii* has been genetically modified to produce the HIV‐1 entry inhibitor, modified cyanovirin‐N (mCV‐N) and resulted in a 63% reduction in HIV transmission when tested in macaques for vaginal colonisation and inhibition of HIV infection (Lagenaur et al., 2011; Lagenaur et al., 2015).

Due to the antimicrobial properties and immunomodulatory effects of *Lactobacillus* spp. (Fan et al., 2021; Taha‐Abdelaziz et al., 2019) these bacteria have also been leveraged as microbial‐derived therapeutic candidates which aim to restore the vaginal microbiota by reducing the abundance of the anaerobic bacterium, *Gardnerella vaginalis* (Ravel et al., 2011). A high abundance of *G. vaginalis* in the female genital tract is associated with poor vaginal health, infections such as bacterial vaginosis (BV) (Ceccarani et al., 2019) and virus‐induced sexual diseases (Severgnini et al., 2022), which can both lead to lower reproductive outcomes (Baud et al., 2023; Guan et al., 2023). The live biotherapeutic, LACTIN‐V (*L. crispatus*) is undergoing Phase2b clinical trials for the treatment for BV (Armstrong et al., 2022); however, the efficacy is dependent on prior antibiotic treatment (Anke et al., 2023). To combat this and reduce antibiotic use, bacteriophages are being investigated as a more targeted therapeutic. Although little is known about bacteriophages within the vaginal microbiome, an association has been found between bacteriophages and BV (Jakobsen et al., 2020). Bioengineered bacteriophages that specifically target *G. vaginalis* (Landlinger et al., 2021) and other disease‐associated bacteria in the genitourinary tract (Miller‐Ensminger et al., 2020) could be harnessed as a new‐generation biotherapeutic. Furthermore, synthetic, rationally designed, microbial consortia that can be delivered as vaginal microbiota transplants (VMTs) are an emerging form of microbial intervention (Lev‐Sagie et al., 2019; Wrønding et al., 2023; Yockey et al., 2022) that can overcome population associated microbiome variation through targeted donor selection.

## MATERNAL HEALTH

Changes in maternal health have impacts on the infant, including on their development or lifelong health. Advances in microbiome analyses have demonstrated associations between the microbiome and women's health (Chen et al., 2017; France et al., 2022; Kaur et al., 2020; Tramice et al., 2022) which are critical modulators of reproductive health (Kaur et al., 2020; Tramice et al., 2022), foetal development (Lundgren et al., 2018) and gestational outcome (Baud et al., 2023; Geldenhuys et al., 2022; Yishay et al., 2023). While the majority of studies that associate pregnancy and pregnancy‐related diseases with the microbiome have been performed in animal models (Gomez de Agüero et al., 2016; Jašarević et al., 2021), a cohort study in South African women that sampled and sequenced bacteria from the gut, vaginal and oral microbiomes found an association with the vaginal microbiome, specifically *Lactobacillus* spp., and the risk of pre‐eclampsia during pregnancy (Geldenhuys et al., 2022). In a broader context, the Women First Preconception Maternal Nutrition Trial identified a decrease in overall bacterial community diversity in the gut of women during pregnancy in LMICs. Specific bacterial taxa were identified as significantly decreased in each country surveyed, including a decrease in *Lachnospiraceae* in Guatemala and *Ruminococcaceae* in Guatemala and Democratic Republic of Congo (Tang et al., 2022). Furthermore, the SHINE trial in Zimbabwe identified key microbial taxa associated with resistant starch‐degradation including *Ruminococcus bromii* and *Faecalibacterium prausnitizii* that are altered during pregnancy and are linked to low birth weight and adverse outcomes for infants (Gough et al., 2021). The bacterial enzymes required for this starch metabolism including glycogen synthase were the most robust predictor of birth weight and gestational age. Within a western population both *R. bromii* and *F. prausnitizii* colonise, but do not correlate with low birth weight and gestational age. It is thought that the lack of *Bacteroides* observed in these mothers may contribute to these alterations in association (Gough et al., 2021). Despite clear progress, the specific consequences for early life development and lifelong health of human gut microbiota in the gestational period remain limited.

## EARLY LIFE MORTALITY

Despite a 44% decrease in neonatal mortality over the past 20 years, it continues to represent an area of greatest disparity between HICs and LMICs (WHO, 2024). During a healthy pregnancy, humans are sterile in utero, with microbial colonisation commencing during birth (de Goffau et al., 2019; Perez‐Muñoz et al., 2017). The first months of life are critical for microbiome acquisition and mark the earliest interactions between the colonising microbiota and the maturing immune system (de Goffau et al., 2019; Di Simone et al., 2023; Donald & Finlay, 2023; Torow & Hornef, 2017). During this time, interactions between the microbiota and innate and adaptive immune systems prime the neonatal immune response (Gollwitzer et al., 2014; Haspeslagh et al., 2018; Kudo et al., 2013). Intrinsic and extrinsic factors may influence microbiome composition including mode of delivery (vaginal or caesarean) (Bosch et al., 2016; Busi et al., 2021; Pattaroni et al., 2018; Shao et al., 2019), breast milk or formula feeding (Biesbroek, Bosch, et al., 2014; Biesbroek, Tsivtsivadze, et al., 2014; Bosch et al., 2017; Shao et al., 2019), gestational age (Amigoni et al., 2017; Ficara et al., 2020; Lao et al., 2022; Neumann et al., 2023; Pattaroni et al., 2018) and antibiotic administration (Gallacher et al., 2020; Keski‐Nisula et al., 2013; Stewart et al., 2018). Furthermore, these communities are then altered through the weaning period where these transition from fermenting to producing acetate when liquid fed, to producing propionate and butyrate when fed solids (Schwab, 2022). These factors together regulate the development of a stable microbial community established over the first 3 years of life (Stewart et al., 2018).

Bacillota (*Streptococcus*, *Staphylococcus*, *Clostridium* and *Lactobacillus*), Pseudomonadota (*Enterobacter*, *Enterococcus* and *Klebsiella*), Actinomycetota (*Bifidobacterium* and *Corynebacterium*) and Bacteroidota (*Bacteroides and Prevotella*) have been reported as early life bacterial colonisers in HICs (Bäckhed et al., 2015; Consortium, 2019; Stewart et al., 2018) with communities shown to vary by country's economic state despite more limited investigation (Harris et al., 2017, 2018; Zimmermann & Curtis, 2018). In general, microbial diversity is lower in microbiomes of children in LMICs, although there is variation between different countries (Chibuye et al., 2024). For example, antibiotic use has been linked to decreased diversity and higher microbial loads of pathogenic *E. coli* strains and antimicrobial resistance genes in microbiomes of children in Mozambique compared to those from the US (Kim et al., 2022; Luchen et al., 2023). Low microbial diversity has been linked to acute and chronic diseases across multiple body sites including asthma (Crow et al., 1975; Haspeslagh et al., 2018; Marri et al., 2013; Raftis et al., 2018), allergy (Gollwitzer et al., 2014; Haspeslagh et al., 2018; Torow & Hornef, 2017), bronchopulmonary dysplasia (Lohmann et al., 2014; Rofael et al., 2019; Viscardi & Hasday, 2009), necrotising enterocolitis (NEC) (Athalye‐Jape et al., 2018; Neumann et al., 2023; Stewart et al., 2016) and IBD (D'Adamo et al., 2023; Gonda et al., 1969). In HICs, factors including household pets and older siblings have been linked to changes in microbial richness and diversity and decreased likelihood of allergic diseases (Azad et al., 2013), but equivalent relationships remain largely untested in LMICs.

In HICs, probiotics, commonly containing *Bifidobacterium* and *Lactobacillus* species, are routinely administered as a preventative treatment to preterm infants born less than 32‐weeks' gestation (Beck et al., 2022). A large cohort study, enrolling 123 preterm infants without gastrointestinal disease or septic infection, showed probiotics were the primary factor shaping bacterial colonisation of the gut microbiome (Beck et al., 2022). Shotgun metagenomic sequencing of 1431 longitudinal stool samples from this cohort identified bacterial communities rich in *Bifidobacterium* species from the probiotic and naturally acquired from the environment. Studies have also shown potential treatment benefits following probiotic administration in reducing the incidence of NEC (Athalye‐Jape et al., 2018; Mercer & Arrieta, 2023; Neumann et al., 2023) and late‐onset sepsis (LOS) (Mercer & Arrieta, 2023) in neonatal and paediatric cohorts. Access to probiotics in LMICs is an important consideration given the increasing numbers of preterm births and incidences of NEC, early‐ and late‐onset sepsis, and risk of pathogenic infection in early life (Deshpande et al., 2017; Unicef, 2024).

Despite numerous large‐scale, early‐life sample cohorts conducted in HICs (Beck et al., 2022; Biesbroek, Tsivtsivadze, et al., 2014; Shao et al., 2019; Stewart et al., 2018), equivalent studies capturing the diversity of conditions across LMICs remain to be completed. Such sampling is essential to guide development of microbiome‐based therapeutics capable of preventing dysbiosis and the associated pathologies, impacting early life in all countries.

## MALNUTRITION

Malnutrition is a major risk factor that impacts maternal and neonatal health; however, it is severely underrepresented in microbiome research (Browne et al., 2024; Desyibelew & Dadi, 2019; Lundgren et al., 2018). The term malnutrition broadly refers to an imbalance of nutrient and energy intake including deficiencies (e.g. undernourishment) or excesses (e.g. obesity). Chronic or recurrent undernourishment is often associated with LMICs, affecting one‐third of countries (Seferidi et al., 2022), and as a result, greatly impacts maternal health and the developing infant (Victora et al., 2021). Germ‐free mice that received a faecal microbiome transplant (FMT) from undernourished infants demonstrated an ameliorated growth rate over 4–5 weeks and significantly reduced weight gain following dietary intervention, compared to those that were colonised with faecal matter from a healthy child (Blanton et al., 2016). Recently, work has begun in developing novel live biotherapeutics in the form of *Prevotella copri* for the treatment of malnutrition (Chang et al., 2024). A randomised controlled trial in malnourished Bangladeshi children identified significant weight gain in children administered a microbiota‐directed complementary food (MDCF) formulation which enhanced the growth of *P. copri* as a therapeutic compared to the control group on the standard nutritional diet (Chang et al., 2024). Furthermore, it was shown that the treatment was able to increase the abundance of *Bifidobacterium infantis* within the gut microbiome of the MDCF cohort, indicating that rationally selected microbiota‐directed formulations can aid the growth of other beneficial bacteria (Chang et al., 2024). Therefore, increasing nutritional intake and probiotic use pose a key therapeutic option, which will directly impact malnutrition, but may have implications for improving future health outcomes.

## VACCINE EFFICACY

The disease prevention potential of vaccines is well established. While higher mortality rates in LMICs are reflective of the reduced vaccination levels, evidence also suggests poorer immunisation responses even in vaccinated individuals (Harris et al., 2017, 2018; Lalor et al., 2009; Zimmermann & Curtis, 2018). For example, Bacillus Calmette‐Guerin (BCG) vaccination protection ranges from 0% to 51% in African infants compared to 88%–100% in European infants (Lalor et al., 2009). Differences in antibody‐specific immune responses have been investigated and associations have been made between the gastrointestinal microbiome and vaccine responsiveness (Harris et al., 2017, 2018; Zimmermann & Curtis, 2018). Bacterial community profiling via 16S rRNA sequencing of 78 infant stool samples from rural Ghana identified differences in microbial community composition, at the genus level, between Rotavirus vaccination responders (*Streptococcus*) and non‐responders (*Bacteroides and Prevotella*) (Harris et al., 2017). Interventional studies using probiotics or antibiotics to enhance vaccine efficacy have been undertaken (Harris et al., 2017, 2018; Zimmermann & Curtis, 2018), where probiotic supplementation using a 4‐strain combination probiotic containing *Bifidobacterium breve*, *Lactobacillus rhamnosus GG*, *Lactobacillus rhamnosus LC705* and *Propionibacterium freudenreichii* improved *Haemophilus influenzae* type b (Hib) vaccine response in 6‐month neonates (Kukkonen et al., 2006). These promising data highlight the need for further research into the potential for optimised microbiome states to improve vaccination response. Such interventions will ensure maximal efficacy of current vaccine interventions and reduce epidemics and cases of infectious diseases in LMICs.

## INFECTIOUS DISEASES

Infectious diseases including HIV/AIDs, malaria, tuberculosis and numerous neglected tropical diseases (NTDs) occur most frequently in LMICs (Chancharoenthana et al., 2022; HIV.gov, 2024; Ippolito et al., 2018; Pescarini et al., 2017). For those diseases where microbiome studies have been undertaken, such as HIV/AIDS, malaria and dengue fever, a significant decrease in microbial diversity in the gut and even the skin microbiota has been observed (Chancharoenthana et al., 2022; Ippolito et al., 2018; Ishizaka et al., 2021; Mukherjee et al., 2022; Vujkovic‐Cvijin et al., 2020). While it remains unclear if a patient microbiome reflects or determines infectious disease state for systemic infections, many diseases are transmitted by an insect vector that also harbour a microbiome. Therefore, examining the insect microbiome for bacterial species linked to decreased transmission or carriage of disease also offers a potential for microbiome‐based therapeutics. A successful use of this technique is in the development of a live biotherapeutic to decrease dengue virus (DENV) transmission and reduce dengue fever. It was shown that *Aedes* mosquito species colonised by the bacterial *Wolbachia* strains *w*MelPop, *w*Mel and *w*AlbB had lower viral transmission and a 50% decrease in mosquito life span (Koh et al., 2019; McMeniman et al., 2009). This strategy is now being deployed globally, where protective efficacies have been reported between 73% and 95% (Fox et al., 2023). Malaria may also represent a target for such interventions (Lee et al., 2023). When the gut microbiomes of mosquitos in hypo‐ and hyper‐endemic regions of South Korea were compared, *Pseudomonas synxantha* was identified as associated with decreased malaria transmission (Lee et al., 2023). There is now a clear need to identify if a causative relationship exists between mosquito carriage of *P. synxantha* and the decreased spread of malaria to guide development of live biotherapeutics for mosquito populations and prevent disease in humans. Importantly deploying this treatment in mosquito populations decreases the need for therapeutic transport and storage in remote locations and comes at no cost to the individuals. In this context, the development of a live biotherapeutic targeting an insect vector, rather than a human host, may provide an optimal point to inhibit disease transmission to a human host and decrease social and economic burdens in LMICs.

## FUTURE OPPORTUNITIES

It is now clear that microbiome‐based therapeutics can be used for disease prevention, treatment and improved efficacy of therapies. These include prophylactic probiotics, increasing the efficacy of conventional treatments and vaccines and targeted live biotherapeutics for both humans and disease vectors such as mosquitos (Gulliver et al., 2022; Schrezenmeir & de Vrese, 2001). The goal for researchers is to fundamentally understand microbial compositions across the diversity of both HIC and LMIC and progress from correlation to causation. These advances will be dependent on appropriate high‐resolution analysis focused on specific taxonomic and phylogenetic microbial clades associated with a specific phenotype of interest. Unlike HIC, the potential use of microbiome‐therapeutics for treatment of diseases prevalent in LMICs remains largely under‐explored. Microbiome optimisation may provide avenues to improve efficacy of existing therapeutics while targeted therapies have the potential to provide new treatment options for existing conditions. In addition to therapeutic efficacy, these treatments can be highly cost‐effective to produce, and the majority of these interventions exhibit long‐term stability at room temperature, removing the need for cold storage and associated supply chain logistical challenges that can pose significant problems in LMICs. Consequently, microbiome‐based therapeutics have immense potential in furthering the goal of improving health and well‐being as a part of the United Nations Sustainable Development Goals and closing the gap between LMICs and HICs.

## AUTHOR CONTRIBUTIONS

**Emily L. Gulliver:** Conceptualization; writing – original draft; writing – review and editing; project administration; supervision. **Sara K. Di Simone:** Writing – original draft; writing – review and editing; conceptualization. **Michelle Chonwerawong:** Writing – original draft; writing – review and editing; conceptualization. **Samuel C. Forster:** Conceptualization; writing – original draft; writing – review and editing; supervision.

## FUNDING INFORMATION

This work is supported by the Australian Research Council (DP190101504) and the Australian National Health and Medical Research Council (APP2000701 and AP1186371). S.C.F is supported by a CSL Centenary Fellowship.

## CONFLICT OF INTEREST STATEMENT

S.C.F. is a scientific advisor to Biomebank Australia. E.L.G, M.C and S.K.D have no conflicts of interest.
